# Supplementary material for: End Sequence Analysis Toolkit (ESAT) expands the extractable information from single-cell RNA-seq data
Source: Genome Res. 2016 Oct;26(10):1397–410. doi: 10.1101/gr.207902.116 (PMC5052061; doi:10.1101/gr.207902.116)
Supplement: Supplemental Material [file supp_gr.207902.116_Supp_Materials.tar.gz › ESAT-esat_v0.1/doc/html/classumms_1_1core_1_1model_1_1score_1_1_scan_statistic_score.html]

ESAT: umms.core.model.score.ScanStatisticScore Class Reference


ESAT


- Main Page
- Related Pages
- Packages
- Classes
- Files

- Class List
- Class Index
- Class Hierarchy
- Class Members

All Classes Namespaces Files Functions Variables Enumerator Pages

- umms
- core
- model
- score
- ScanStatisticScore

Classes |
Public Member Functions |
List of all members

umms.core.model.score.ScanStatisticScore Class Reference

Inheritance diagram for umms.core.model.score.ScanStatisticScore:

[legend]

Collaboration diagram for umms.core.model.score.ScanStatisticScore:

[legend]

|  |  |
| --- | --- |
| Classes | |
| class | **Processor** |
|  | |

|  |  |
| --- | --- |
| Public Member Functions | |
|  | ScanStatisticScore (AlignmentModel model, Annotation annotation) |
|  | |
|  | ScanStatisticScore (AlignmentModel model, Annotation annotation, boolean fullyContained) |
|  | |
|  | ScanStatisticScore (AlignmentModel model, Annotation annotation, ScanStatisticScore previousScore, double newScore) |
|  | |
|  | ScanStatisticScore (MultiScore other) |
|  | |
|  | ScanStatisticScore (AlignmentModel model, Annotation annotation, double regionTotal, double regionLength) |
|  | |
|  | ScanStatisticScore (AlignmentModel model, Annotation annotation, double regionTotal, double regionLength, boolean fullyContained) |
|  | |
|  | ScanStatisticScore (AlignmentModel model, Annotation annotation, double regionTotal, double regionLength, double globalTotal, double globalLength, boolean fullyContained) |
|  | |
| CoordinateSpace | getCoordinateSpace () |
|  | |
| void | refreshScanPvalue (AlignmentModel model) |
|  | |
| double | getAverageCoverage (AlignmentModel data) |
|  | |
| double | getEnrichment (AlignmentModel data) |
|  | |
| double | getLocalLambda () |
|  | |
| void | setGlobalLength (double d) |
|  | |
| double | getGlobalLambda () |
|  | |
| double | getGlobalLength () |
|  | |
| void | setRegionLength (double regionLength) |
|  | |
| double | getRegionLength () |
|  | |
| void | setScanPvalue (double scanPvalue) |
|  | |
| void | setPvalue (double scanPvalue) |
|  | |
| double | getScanPvalue () |
|  | |
| double | getPvalue () |
|  | |
| void | setFullyContainedNumberOfReads (double fullyContainedNumberOfReads) |
|  | |
| double | getFullyContainedNumberOfReads () |
|  | |
| double[] | getScores () |
|  | |
| void | getDebugInfo (String prefix) |
|  | |
| String | toString () |
|  | |
| boolean | equals (Object o) |
|  | |
| Public Member Functions inherited from umms.core.model.score.CountScore | |
|  | CountScore (Annotation a) |
|  | |
|  | CountScore (CountScore other) |
|  | |
|  | CountScore (CountScore other, Annotation newAnnotation, double newCount) |
|  | |
|  | CountScore (AnnotationCollection<?extends Annotation > model, Annotation annotation) |
|  | |
|  | CountScore (AnnotationCollection<?extends Annotation > model, Annotation annotation, double regionTotal) |
|  | |
|  | CountScore (AnnotationCollection<?extends Annotation > model, Annotation annotation, boolean fullyContained) |
|  | |
|  | CountScore (AnnotationCollection<?extends Annotation > model, Annotation annotation, double regionTotal, boolean fullyContained) |
|  | |
|  | CountScore (Annotation annotation, double count, double regionTotal, double total) |
|  | |
| double | getCount () |
|  | |
| double | getScore () |
|  | |
| double | getTotal () |
|  | |
| double | getRegionTotal () |
|  | |
| double | getRPKM () |
|  | |
| double | getRPK () |
|  | |
| void | setCount (double count) |
|  | |
| void | setTotal (double total) |
|  | |
| void | setRegionTotal (double regionTotal) |
|  | |
| String | toString () |
|  | |
| boolean | equals (Object o) |
|  | |
| int | compareTo (CountScore o) |
|  | |
| int | hashCode () |
|  | |
| void | refreshPvalue () |
|  | |
| double | getPvalue () |
|  | |
| Public Member Functions inherited from umms.core.model.score.WindowScore.AbstractWindowScore | |
|  | AbstractWindowScore (Annotation t) |
|  | |
| Annotation | getAnnotation () |
|  | |

|  |  |
| --- | --- |
| Additional Inherited Members | |
| Static Public Member Functions inherited from umms.core.model.score.CountScore | |
| static double | asRPKM (double count, double total, int windowSize) |
|  | |
| Static Public Attributes inherited from umms.core.model.score.CountScore | |
| static double | DEFAULT\_REGION\_TOTAL = -1.0 |
|  | |
| Protected Attributes inherited from umms.core.model.score.WindowScore.AbstractWindowScore | |
| Annotation | annotation |
|  | |

## Constructor & Destructor Documentation

|  |  |  |  |
| --- | --- | --- | --- |
| umms.core.model.score.ScanStatisticScore.ScanStatisticScore | ( | AlignmentModel | *model*, |
|  |  | Annotation | *annotation* |
|  | ) |  |  |

Here is the caller graph for this function:

|  |  |  |  |
| --- | --- | --- | --- |
| umms.core.model.score.ScanStatisticScore.ScanStatisticScore | ( | AlignmentModel | *model*, |
|  |  | Annotation | *annotation*, |
|  |  | boolean | *fullyContained* |
|  | ) |  |  |

Here is the call graph for this function:

|  |  |  |  |
| --- | --- | --- | --- |
| umms.core.model.score.ScanStatisticScore.ScanStatisticScore | ( | AlignmentModel | *model*, |
|  |  | Annotation | *annotation*, |
|  |  | ScanStatisticScore | *previousScore*, |
|  |  | double | *newScore* |
|  | ) |  |  |

Here is the call graph for this function:

|  |  |  |  |  |  |
| --- | --- | --- | --- | --- | --- |
| umms.core.model.score.ScanStatisticScore.ScanStatisticScore | ( | MultiScore | *other* | ) |  |

Here is the call graph for this function:

|  |  |  |  |
| --- | --- | --- | --- |
| umms.core.model.score.ScanStatisticScore.ScanStatisticScore | ( | AlignmentModel | *model*, |
|  |  | Annotation | *annotation*, |
|  |  | double | *regionTotal*, |
|  |  | double | *regionLength* |
|  | ) |  |  |

|  |  |  |  |
| --- | --- | --- | --- |
| umms.core.model.score.ScanStatisticScore.ScanStatisticScore | ( | AlignmentModel | *model*, |
|  |  | Annotation | *annotation*, |
|  |  | double | *regionTotal*, |
|  |  | double | *regionLength*, |
|  |  | boolean | *fullyContained* |
|  | ) |  |  |

Here is the call graph for this function:

|  |  |  |  |
| --- | --- | --- | --- |
| umms.core.model.score.ScanStatisticScore.ScanStatisticScore | ( | AlignmentModel | *model*, |
|  |  | Annotation | *annotation*, |
|  |  | double | *regionTotal*, |
|  |  | double | *regionLength*, |
|  |  | double | *globalTotal*, |
|  |  | double | *globalLength*, |
|  |  | boolean | *fullyContained* |
|  | ) |  |  |

Control region length and total, global length and total Scan P value depends on global stats

Parameters
:   |  |  |
    | --- | --- |
    | model | Alignment data |
    | annotation | Annotation |
    | regionTotal | Total number of fragments mapping to local region |
    | regionLength | Total length of local region |
    | globalTotal | Global number of fragments |
    | globalLength | Global length |

Here is the call graph for this function:

## Member Function Documentation

|  |  |  |  |  |  |
| --- | --- | --- | --- | --- | --- |
| boolean umms.core.model.score.ScanStatisticScore.equals | ( | Object | *o* | ) |  |

True iff scan P value and annotation are equal

Here is the call graph for this function:

|  |  |  |  |  |  |
| --- | --- | --- | --- | --- | --- |
| double umms.core.model.score.ScanStatisticScore.getAverageCoverage | ( | AlignmentModel | *data* | ) |  |

Here is the call graph for this function:

Here is the caller graph for this function:

|  |  |  |  |  |
| --- | --- | --- | --- | --- |
| CoordinateSpace umms.core.model.score.ScanStatisticScore.getCoordinateSpace | ( |  | ) |  |

Here is the caller graph for this function:

|  |  |  |  |  |  |
| --- | --- | --- | --- | --- | --- |
| void umms.core.model.score.ScanStatisticScore.getDebugInfo | ( | String | *prefix* | ) |  |

Here is the call graph for this function:

|  |  |  |  |  |  |
| --- | --- | --- | --- | --- | --- |
| double umms.core.model.score.ScanStatisticScore.getEnrichment | ( | AlignmentModel | *data* | ) |  |

Here is the call graph for this function:

|  |  |  |  |  |
| --- | --- | --- | --- | --- |
| double umms.core.model.score.ScanStatisticScore.getFullyContainedNumberOfReads | ( |  | ) |  |

|  |  |  |  |  |
| --- | --- | --- | --- | --- |
| double umms.core.model.score.ScanStatisticScore.getGlobalLambda | ( |  | ) |  |

Here is the call graph for this function:

Here is the caller graph for this function:

|  |  |  |  |  |
| --- | --- | --- | --- | --- |
| double umms.core.model.score.ScanStatisticScore.getGlobalLength | ( |  | ) |  |

Here is the caller graph for this function:

|  |  |  |  |  |
| --- | --- | --- | --- | --- |
| double umms.core.model.score.ScanStatisticScore.getLocalLambda | ( |  | ) |  |

Here is the call graph for this function:

Here is the caller graph for this function:

|  |  |  |  |  |
| --- | --- | --- | --- | --- |
| double umms.core.model.score.ScanStatisticScore.getPvalue | ( |  | ) |  |

|  |  |  |  |  |
| --- | --- | --- | --- | --- |
| double umms.core.model.score.ScanStatisticScore.getRegionLength | ( |  | ) |  |

Here is the caller graph for this function:

|  |  |  |  |  |
| --- | --- | --- | --- | --- |
| double umms.core.model.score.ScanStatisticScore.getScanPvalue | ( |  | ) |  |

Here is the caller graph for this function:

|  |  |  |  |  |
| --- | --- | --- | --- | --- |
| double [] umms.core.model.score.ScanStatisticScore.getScores | ( |  | ) |  |

Here is the call graph for this function:

|  |  |  |  |  |  |
| --- | --- | --- | --- | --- | --- |
| void umms.core.model.score.ScanStatisticScore.refreshScanPvalue | ( | AlignmentModel | *model* | ) |  |

Here is the call graph for this function:

Here is the caller graph for this function:

|  |  |  |  |  |  |
| --- | --- | --- | --- | --- | --- |
| void umms.core.model.score.ScanStatisticScore.setFullyContainedNumberOfReads | ( | double | *fullyContainedNumberOfReads* | ) |  |

|  |  |  |  |  |  |
| --- | --- | --- | --- | --- | --- |
| void umms.core.model.score.ScanStatisticScore.setGlobalLength | ( | double | *d* | ) |  |

Here is the caller graph for this function:

|  |  |  |  |  |  |
| --- | --- | --- | --- | --- | --- |
| void umms.core.model.score.ScanStatisticScore.setPvalue | ( | double | *scanPvalue* | ) |  |

|  |  |  |  |  |  |
| --- | --- | --- | --- | --- | --- |
| void umms.core.model.score.ScanStatisticScore.setRegionLength | ( | double | *regionLength* | ) |  |

Here is the caller graph for this function:

|  |  |  |  |  |  |
| --- | --- | --- | --- | --- | --- |
| void umms.core.model.score.ScanStatisticScore.setScanPvalue | ( | double | *scanPvalue* | ) |  |

Here is the caller graph for this function:

|  |  |  |  |  |
| --- | --- | --- | --- | --- |
| String umms.core.model.score.ScanStatisticScore.toString | ( |  | ) |  |

Here is the call graph for this function:

---

The documentation for this class was generated from the following file:

- java/umms/core/model/score/ScanStatisticScore.java


---

Generated on Fri Aug 15 2014 15:11:13 for ESAT by  

 1.8.7
